# Regulation of the Adaptive Immune Response by the IκB Family Protein Bcl-3

**DOI:** 10.3390/cells5020014

**Published:** 2016-03-24

**Authors:** Felicity D. Herrington, Robert J. B. Nibbs

**Affiliations:** Institute of Infection, Immunity and Inflammation, University of Glasgow, Glasgow G12 8TA, Scotland, UK

**Keywords:** NF-κB, Bcl-3, adaptive immunity, inflammation

## Abstract

Bcl-3 is a member of the IκB family of proteins and an important regulator of Nuclear Factor (NF)-κB activity. The ability of Bcl-3 to bind and regulate specific NF-κB dimers has been studied in great depth, but its physiological roles *in vivo* are still not fully understood. It is, however, becoming clear that Bcl-3 is essential for the proper development, survival and activity of adaptive immune cells. Bcl-3 dysregulation can be observed in a number of autoimmune pathologies, and *Bcl3*-deficient animals are more susceptible to bacterial and parasitic infection. This review will describe our current understanding of the roles played by Bcl-3 in the development and regulation of the adaptive immune response, including lymphoid organogenesis, immune tolerance, lymphocyte function and dendritic cell biology.

## 1. NF-κB

The NF-κB family of transcription factors control the transcription of hundreds of genes and regulate many key biological processes [1]. The majority of NF-κB target genes encode immunomodulatory proteins, including cytokines [2,3,4], chemokines [5], and proteins involved in the presentation of antigen to immune cells (www.nf-kb.org). Consequently, NF-κB is essential for the development of the immune system and the induction of successful immunity [6,7,8], playing indispensable roles in both the initiation and contraction of responses. NF-κB is therefore widely considered to be a master regulator of the immune response.

NF-κB is a heterogeneous collection of homo- and heterodimers, with five proteins making up the mammalian NF-κB family: p50, p52, p65 (RelA), c-Rel, and RelB. All share a conserved ~300 amino-acid N-terminal Rel homology domain, which enables these NF-κB subunits to dimerise, localize to the nucleus, and bind DNA [9]. p50 and p52 differ from the other NF-κB subunits in that they are synthesized as large precursor proteins (p105 and p100, respectively) that must be processed by the proteasome to generate mature subunits. The p100 and p105 precursors also play important regulatory functions in the activation of NF-κB dimers via their C terminal ankyrin repeat domains (see below). Functional p50 and p52 lack the C-terminal transactivation domain found in the other NF-κB subunits. Consequently, p50 and p52 homodimers do not possess intrinsic transactivational activity [10], and are generally considered to be repressors of gene transcription. However, p50 and p52 can also hetero-dimerise with other NF-κB subunits, with the resulting heterodimers able to activate transcription.

Two separate signaling pathways result in the activation of distinct dimeric NF-κB species, leading to different biological outcomes. Activation of the classical (or canonical) pathway results predominantly in the nuclear translocation of dimers containing c-Rel, p65 and/or p50. This pathway is primarily involved in mounting effective immune responses, and contributes to the control of cell proliferation and survival. The second pathway is the non-canonical, or alternative pathway. Activation of the non-canonical pathway is slower than that of the classical pathway, and centres on the activation of the RelB and p52 subunits, controlling genes important in the regulation of homeostatic processes [11]. It occurs downstream of a discrete group of receptors belonging to the tumor necrosis factor receptor superfamily, including the lymphotoxin-β receptor, CD40, and the B-cell activating factor (BAFF) receptor. It is worth noting, however, that certain receptors can activate both pathways, and that considerable crosstalk exists between the signaling pathways [12].

The aberrant activation of NF-κB-mediated transcription is associated with various pathologies, and can inflict severe damage to host tissues. As such, the regulation of NF-κB activity is essential, and tightly controlled [13]. The principal means of restraining NF-κB activation is through the interaction of NF-κB dimers with inhibitor of κB (IκB) proteins. The IκB family share a homologous central domain comprised of 5–7 ankyrin repeats, which mediate their interactions with NF-κB dimers [14]. These proteins can be further classified into three subfamilies: the prototypical IκBs (IκBα, IκBβ, and IκBε); the atypical IκBs (Bcl-3 [15], IκBNS [16], IκBζ [17] and IκBη [18]); and the p50 and p52 precursors, p105 and p100, respectively. The prototypical IκB molecules, along with p105 and p100, interact with NF-κB dimers in the cytoplasm, inhibiting their translocation to the nucleus. On receipt of an activatory stimulus, ubiquitin-dependent degradation of these IκB proteins occurs, liberating active NF-κB dimers, which translocate into the nucleus and can then bind DNA target sequences. The atypical IκB molecules, in contrast, are not degraded following NF-κB pathway activation and reside primarily in the nucleus where they interact with NF-κB dimers to regulate transcription. This modulation of NF-κB-dependent transcriptional programs is context-dependent, occurring in response to specific stimuli received by the cell, and is essential for successful, controlled immune responses.

## 2. Bcl-3

The most extensively studied atypical IκB molecule, Bcl-3, was first identified as a putative proto-oncogene. Cloning of chromosomal breakpoints found in chronic lymphocytic leukemia cells demonstrated that the t(14;19) chromosomal translocation results in the juxtaposition of the *BCL3* gene with the immunoglobulin (Ig) heavy locus leading to the overexpression of Bcl-3 in leukemic cells [19,20,21]. Bcl-3 overexpression is also associated with a number of cancers that do not harbor *BCL3*-associated translocations, including classical Hodgkin’s and peripheral T-cell lymphoma [22], chronic lymphocytic leukemia [23], adult T cell leukemia [24], and certain solid tumors [25,26].

The Bcl-3 protein contains a central domain comprised of 7 ankyrin-repeats, flanked by a proline-rich N terminal region and a serine/proline-rich C terminal region [27]. All IκB proteins contain ankyrin-repeat domains, and the structural similarity between this region of Bcl-3 and previously identified IκB molecules led to the prediction that Bcl-3 would exhibit NF-κB-regulatory functions. Indeed, further studies identified Bcl-3 as an IκB protein [15,28,29] and demonstrated its ability to interact with the p50 and p52 subunits of NF-κB via its ankyrin repeat domain [15,30,31,32]. Early reports, however, disagreed on the precise role of Bcl-3 within the NF-κB pathways, and it was proposed that Bcl-3 was able to: (i) block the nuclear translocation of p50 [33,34]; (ii) inhibit DNA-binding of p50 homo- and heterodimers [30,32,33]; (iii) limit p50-mediated NF-κB repression [31]; (iv) remove bound p50-containing dimers from DNA [35]; (v) induce cytoplasmic activation of p50 [34]; and (vi) directly transactivate genes when complexed with p50 [27,36] or p52 [37]. Nonetheless, it is now generally accepted that Bcl-3 regulates classical and non-canonical NF-κB-dependent gene transcription through selective interaction with homodimers of p50 and p52 [28,30,32,38], without inhibiting DNA-binding. In fact, it has been shown in thymocytes that Bcl-3 enhances binding of p50 homodimers to NF-κB target sites in DNA [39]. Additionally, studies using bone marrow-derived macrophages demonstrate that Bcl-3 stabilizes DNA-bound p50 homodimers through the inhibition of p50 ubiquitination and proteasomal degradation [40]. In response to Toll-like receptor and tumor necrosis factor (TNF) receptor signalling, κB target sites in the DNA can become blocked by Bcl-3-stabilized p50 homodimers, preventing transcriptionally active dimers from binding and driving gene transcription [40]. In this way, Bcl-3 is able to limit the pro-inflammatory transcriptional programs initiated by certain classical pathway stimuli [40,41]. However, Bcl-3-mediated suppression of the classical pathway is likely to be context and/or stimulus dependent, and further work is still needed to fully understand the functional consequences of the interaction of Bcl-3 with p50 homodimers.

The role of Bcl-3 in the regulation of p52 homodimers is even less clear. Evidence points to a requirement of Bcl-3 for optimal non-canonical signaling [42], with the phenotypes of *Bcl3* knockout mice (*Bcl3*^−/−^) echoing those of mice with defects in specific non-canonical NF-κB signaling components [43,44]. Interestingly, Bcl-3 has also been reported to act as an adaptor molecule, capable of recruiting a number of nuclear coactivator and corepressor complexes to p50/52 homodimers [45], and itself contains two transactivating domains that are able to directly stimulate transcription when bound to homodimers of p50 or p52 [27,36]. Clearly, these non-IκB activities of Bcl-3 add a further layer of complexity to the potential functions of this protein, especially in the context of Bcl-3:p50 homodimer interaction. Bcl-3 has a predominantly repressive role in the classical pathway of NF-κB activation, however under certain circumstances it may also act to enhance the transcription of specific genes [46]. The molecular basis for the dual function of Bcl-3 in the classical pathway, and the signals that control its inhibitory *versus* activatory potential, are still not fully understood.

Unravelling the molecular details of Bcl-3 is further complicated by its numerous post-translational modifications (PTMs). Bcl-3 is a highly phosphorylated protein [27,32,39,47], with phosphorylation at specific sites shown to be crucial for its activity in certain contexts. Phosphorylation of Bcl-3 by the protein kinase GSK3 selectively regulates the ability of Bcl-3 to control transcription of a subset of NF-κB target genes [37]. Microarray analysis of NIH3T3 cells transfected with either wild-type Bcl-3 or a Bcl-3 mutant lacking GSK phosphorylation sites demonstrated the differential regulation of *Slpi*, *Cxcl1*, *Ifi205* and *Cypibi* by phosphorylated and un-phosphorylated Bcl-3 [37]. Hypo-phosphorylated Bcl-3 has been shown to have increased interaction with transcriptional corepressors [37], and studies looking at nuclear extracts from Bcl-3 transgenic thymocytes have shown that Bcl-3 de-phosphorylation lessens its ability to enhance DNA:p50 homodimer binding [39]. Ubiquitination of Bcl-3 also plays a key role in its activation by regulating intracellular Bcl-3 localization. Although primarily located in the nucleus, in certain cell types inactive Bcl-3 localizes to the cytoplasm [48,49]. Cytoplasmic Bcl-3 requires K63-linked polyubiquitination in order to translocate to the nucleus. The de-ubiquitinase CYLD has been shown to control Bcl-3 localization in keratinocytes through the removal of these polyubiquitin chains, preventing nuclear accumulation of Bcl-3 and consequently, Bcl-3-mediated regulation of gene transcription [50]. It is not yet fully understood how these, and other, PTMs affect Bcl-3 function, but they may act as a route through which cellular responses can be precisely manipulated, depending on the particular cell type and stimulus received.

**Figure 1:**
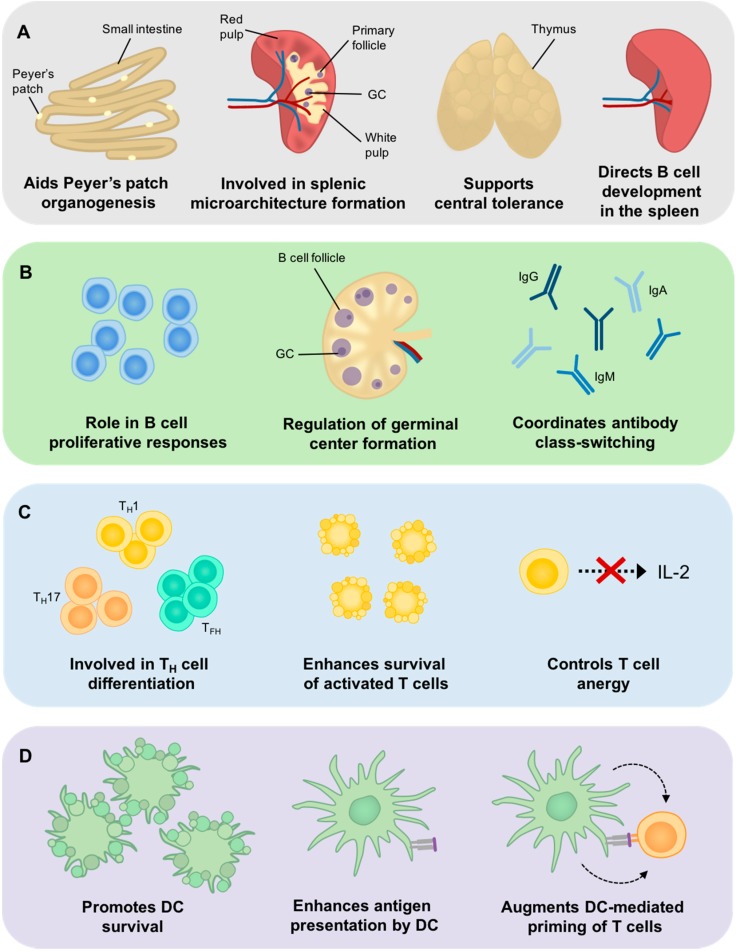
Bcl-3 is involved in the development and function of the adaptive immune response. (**A**) Bcl-3 is an important factor in the development of the adaptive immune system, with roles in the organogenesis of secondary lymphoid organs (SLOs), such as Peyer’s patches, the correct formation of splenic microarchitecture, the induction of central T cell tolerance in the thymus, and the development of lymphocytes within SLOs; (**B**) Bcl-3 is crucial to the regulation of B cell proliferative responses, effective germinal center (GC) formation and the production of class-switched antibodies; (**C**) Bcl-3 is involved in the differentiation and development of T_H_ cell subsets in the periphery, acts as a survival factor in activated T cells, and has been implicated in the induction of anergy in CD4^+^ T cells; (**D**) Bcl-3 promotes DC survival, and enhances successful antigen presentation and T cell priming by DCs.

Although the molecular characterization of Bcl-3 has revealed several important mechanisms through which NF-κB activity may be controlled, much is still to be uncovered. Along with work aimed at defining the molecular details of Bcl-3, many studies have focused on understanding the cellular functions of Bcl-3 *in vivo*. Bcl-3’s involvement in the immune system has been examined in some depth, and this review will focus on the adaptive immune response, exploring the current knowledge in this field (summarized in Figure 1). It is worth noting that Bcl-3 has also been implicated in the regulation of multiple innate immune cell populations, including macrophages [40,51,52,53], neutrophils [54], mast cells [55], and certain stromal cells [56], however these are outwith the scope of this review, and so will not be discussed further.

To aid readers who are not immunologists, we will first provide an overview of the adaptive immune system in the hope that this will make our subsequent descriptions of Bcl-3 function more broadly accessible. Those with an immunology background may wish to skip this part of the review and go straight to Section 4.

## 3. The Adaptive Immune Response

The immune system is comprised of two interacting branches: the innate immune system, providing rapid first line defense against infection; and the adaptive immune system, which offers a slower, but more tailored response and generates immunological memory. Adaptive immune responses require the presentation of antigens (Ags) to lymphocytes expressing specific cell-surface Ag receptors. There are two major lymphocyte populations: T cells and B cells. Each lymphocyte carries a unique Ag receptor, referred to as the T Cell Receptor (TCR) or the B Cell Receptor (BCR), and consequently, lymphocyte populations are capable of recognizing an enormous number of Ags.

Lymphocytes develop from haemopoietic stem cells in the bone marrow. T cells undergo further development in the thymus, while B cells complete their maturation in the spleen. During development, each lymphocyte expresses a randomly generated TCR or BCR on its surface. Many of these are non-functional or recognize self-Ags. To eliminate cells carrying such receptors, immature lymphocytes undergo a screening process, referred to as central tolerance. This occurs in the bone marrow for B cells [57], and in the thymus for T cells [58], and is important as the release of self-reactive lymphocytes into the periphery can lead to the development of autoimmunity. During T cell development, a specialized population of immunosuppressive regulatory T cells (T_regs_) are also generated, called natural T_regs_, which can suppress autoimmunity and regulate adaptive immune responses [59]. These developmental processes generate the pool of circulating “naïve” lymphocytes. Naïve T cells express either the CD4 co-receptor, and are referred to as CD4^+^ T cells or T helper cells, or the CD8 co-receptor, in which case they are called CD8^+^ T cells or cytotoxic T cells [60]. Most naïve B cells are known as follicular B cells (FO B cells), but two rare populations, marginal zone B cells (MZ B cells) and B1 B cells, are also found in mice.

To participate in adaptive immunity, naïve lymphocytes must encounter their cognate Ag. This occurs primarily in the specialised microenvironments of secondary lymphoid organs (SLOs) (e.g., spleen, lymph nodes (LNs), tonsils, and intestinal Peyer’s patches)**.** Each SLO efficiently captures and presents Ags from particular extracellular fluids: for example, the spleen filters blood while LNs trap Ags in lymph. In SLOs, B cells and T cells segregate into specific microanatomical niches containing distinct Ag-presenting cells and stromal cells. FO B cells reside in follicles, MZ B cells in the splenic marginal zone, and T cells in T cell areas. It is here that naïve lymphocytes encounter Ag, and where adaptive immune responses are initiated and predominantly regulated.

Naïve T cells respond to Ag presented on major histocompatibility complex (MHC) proteins by dendritic cells (DCs) in the periphery [61]. DCs also provide information to Ag-specific T cells that dictate their subsequent fate. Clearly it is not desirable for T cells to respond to self-Ags or harmless Ags in food or commensal bacteria, so these Ags are presented in such a way that any T cells that recognise them are either destroyed, converted into induced T_regs_, or made anergic (*i.e.*, become functionally unresponsiveness to Ag). This process is referred to as peripheral tolerance, and is a second layer of T cell regulation, following central tolerance in the thymus. In contrast, it is essential that T cells that recognise Ag from a potentially harmful pathogen are instructed to contribute to immune defence. DCs presenting such Ags send signals to T cells that drive their proliferation (to increase the number of Ag-specific T cells), and push them to develop functional properties that aid pathogen clearance. CD8^+^ T cells develop the ability to kill malignant or infected cells, while activated CD4^+^ T cells can differentiate into a number of different types of T helper cell (e.g., T_H_1, T_H_2, T_H_17, or T_FH_) [62]. Each T helper cell subset expresses a characteristic repertoire of immunomodulatory cytokines and can influence specific aspects of the immune response, such as inflammation, cytotoxicity, or antibody (Ab) production by B cells (see below). Moreover, some activated T cells develop into memory T cells that are far more responsive to Ag than naïve T cells, and mount rapid and robust responses when Ag-bearing pathogens are encountered again. Multiple checks and balances exist to ensure that T cell responses are controlled and resolved: T_regs_ play a critical role by suppressing activated T cells via direct cell contact and immunosuppressive cytokine release.

The principal purpose of B cells is to develop into Ab-secreting cells (ASCs). B cells can be activated by cognate Ag in several ways, each leading to their proliferation and differentiation into ASCs. However, without “help” from follicular T helper cells (T_FH_), these ASCs are short-lived, and the Ab they release has relatively low affinity for Ag and limited effector functions. To receive help, B cells present peptides derived from their cognate Ag on MHC to T_FH_ cells previously activated by the same Ag presented on MHC by DCs. T_FH_ cells support the development of structures in SLOs called Germinal Centers (GCs) in which activated B cells undergo further differentiation and selection [63]. The ASCs formed in GCs have an extended lifespan and produce high affinity Abs, and, because of a process called “class-switching”, these Abs have a broader range of effector functions. GCs also generate memory B cells, which rapidly produce high affinity Abs when their cognate Ag is re-encountered. Since powerful B cell responses depend on activated T cells, and T cells are subject to the regulation described above, unwelcome and potentially damaging B cell responses are indirectly avoided.

In summary, the adaptive immune system, via the coordinated interaction of DCs, T cells and B cells in specialised tissues, delivers a flexible controlled response to pathogen-associated Ags and generates memory cells for lifelong protection. Although the existence of autoimmunity demonstrates its fallibility, adaptive immunity is essential for human life and the target of effective vaccines.

## 4. Bcl-3 Involvement in Central Tolerance

Medullary thymic epithelial cells (mTECs) are stromal cells in the thymus critical for effective central tolerance [64]: they express and present Ags found elsewhere in the body. T cells are exposed to these Ags whilst in the thymus, and any potentially auto-reactive T cells that recognize these Ags are deleted [65]. Defective mTEC development or function can lead to multiorgan inflammation due to the release of auto-reactive T cells into the periphery. Mice deficient in either *Nfkb2* (which encodes p52/p100) or *Bcl3* demonstrate no overt autoimmune pathology, however mice lacking both genes (*Nfkb2*^−/−^*Bcl3*^−/−^) spontaneously develop inflammatory infiltrates in multiple organs and die within four weeks of birth [43]. While natural T_reg_ development and function appears normal in *Nfkb2*^−/−^*Bcl3*^−/−^ mice, these animals have increased numbers of activated auto-reactive T cells [43]. Moreover, autoimmune pathology can be induced by transferring *Nfkb2*^−/−^*Bcl3*^−/−^ T cells into *Rag1*^−/−^ mice (which do not produce their own T or B cells) [43]. Thus, auto-reactive T cells drive inflammation in *Nfkb2*^−/−^*Bcl3*^−/−^ animals, pointing to a defect in central tolerance. Studies using bone-marrow chimeras indicate that stromal cells, rather than haemopoietic cells, are necessary and sufficient for the development of autoimmunity in these mice. They have a highly abnormal thymus, which has virtually disappeared by four weeks of age. Moreover, mTEC development is defective and this is likely to be major reason why these mice are unable to successfully eliminate auto-reactive T cells. Although the molecular basis for this defect has not been precisely defined, *Nfkb2* deficiency removes p52, so the impact of *Bcl3* deletion in mice lacking *Nfkb2* is likely to be due to alterations in classical NF-κB signalling stemming from the loss of p50/Bcl-3 interactions. Based on these findings, it appears that activation of both NF-κB pathways is required to develop fully functional mTEC and/or other stromal cells involved in central tolerance, although further studies are required to determine precisely how the NF-κB pathways are working in these cells.

## 5. The Role of Bcl-3 in SLO Development

It has long been known that NF-κB plays a critical role in the development of SLOs [44], and so it is not surprising that *Bcl3* deficiency also leads to developmental defects in SLOs. *Bcl3*^−/−^ mice have impaired Peyer’s patch development, as well as disruptions in their splenic and LN architecture. Peyer’s patches are macroscopic aggregated lymphoid follicles found at regular intervals along the length of the small intestine. Large B cell follicles sit adjacent to a T cell-rich area and are separated from specialized follicle-associated epithelium by the DC-rich sub-epithelial dome (SED), which captures Ags delivered from the gut lumen [66]. Compared to wild-type mice, Peyer’s patches in *Bcl3*^−/−^ mice are smaller and less abundant, although a far stronger phenotype is seen in mice lacking either *Nfkb1* (which encodes p50/p105) or *Nfkb2* [38]. The Peyer’s patches that do develop in *Bcl3*^−/−^ mice have enlarged T cell areas and small follicles, which contain fewer follicular dendritic cells (FDCs), an antigen-presenting stromal cell present in SLO follicles [38]. The distribution of DCs in the SED is also abnormal in these animals, and there is an increase in the number of M cells, specialized epithelial cells that transport Ags from the gut lumen to the SED [38]. Interestingly, the expression of the chemokines CXCL13 and CCL20, which drive the recruitment of cells to follicles and SED, respectively, are highly dependent on p100/p52 and Bcl-3 [38], which may explain the defects in the Peyer’s patches of *Bcl3*^−/−^ mice. This could also account for the reduction in Peyer’s patch number, as CXCL13 plays a critical role in inducing Peyer’s patch formation through its ability to recruit lymphoid tissue inducer cells to the wall of the small intestine during embryogenesis [67,68].

The lymph nodes of *Bcl3*^−/−^ mice are reported to be hypoplastic and, like the Peyer’s patches, contain fewer B cells. Additionally, the spleens of these animals contains abnormally small follicles containing fewer B cells, FDCs and GCs [44,69,70]. The non-canonical NF-κB pathway is known to be crucial for the correct formation and function of SLOs, and the phenotypic similarities between *Nfkb2*^−/−^ mice and *Bcl3*^−/−^ mice have led to the proposition that Bcl-3 enhances p52-mediated transcription during lymphoid organogenesis. However, as in the thymus, *Bcl3* deficiency substantially enhances SLO phenotypes in *Nfkb2*^−/−^ mice: while *Nfkb2*^−/−^ and *Bcl3*^−/−^ mice have lymph nodes, *Nfkb2*^−/−^*Bcl3*^−/−^ mice completely lack these organs [71]. As such, it seems likely that *Bcl3* deficiency leads to alterations in p50 function or regulation during embryogenesis. However, these observations do not exclude the possibility that SLO defects in mice lacking only *Bcl3* are caused, at least in part, by dysregulation of the non-canonical NF-κB pathway.

## 6. The Role of Bcl-3 in B Cell Development and Function

The most obvious phenotype in *Bcl3*^−/−^ animals is the reduction in the number of B cells in their SLOs. Interestingly, it is actually only FO B cell numbers that are reduced: these animals have more MZ B cells than wild-type mice [71]. MZ B cells are a sessile population of cells that reside in the splenic MZ and participate in T cell-independent B cell responses to blood-borne Ag/pathogens [72]. In mice, the final steps of B cell development occur in the spleen, with newly arriving bone marrow-derived immature B cells passing through a series of transitional stages before differentiating into either FO or MZ B cells. Cell fate decisions depend on the strength of specific intracellular signals, and the deletion of genes that regulate these signals often alters the FO/MZ B cell ratio [73]. The canonical NF-κB pathway transduces one of these key fate-determining signals, and so the B cell phenotype in *Bcl3*^−/−^ mice likely reflects a change in the activity of this pathway. Indeed, in support of this hypothesis, Zhang and colleagues have demonstrated that this phenotype is due to a B cell-autonomous defect, rather than an aberration in the splenic stromal cell compartment [71]. However, the molecular mechanisms by which Bcl-3 regulates MZ B cell development remain to be defined, although a modest increase in integrin expression by *Bcl3*^−/−^ MZ B cells might aid their retention in the MZ and contribute to their increased abundance in the spleen [71]. Also, it is worth noting that the splenic MZ of *Bcl3*^−/−^ mice contains fewer macrophages than wild-type mice and has certain molecular defects, including reduced expression of MAdCAM-1 by sinus-lining cells and lower levels of the basement membrane protein laminin β-2 adjacent to these cells [69,70]. Again, the molecular basis for these defects are not known, and it is not clear whether these features contribute to, or are a consequence of, the accumulation of MZ B cells in these animals.

Several different mouse strains have been generated that over-express Bcl-3 in their B cell compartments. These mice have been used to examine the oncogenic functions of Bcl-3, but their immunological phenotypes are also of interest. Eµ-*BCL3* mice express a human *BCL3* transgene in both their T and B cells [74], while two recently-developed strains, including Bcl-3^BOE^ mice, carry a B cell-restricted mouse *Bcl3* transgene [71,75]. In all of these strains there is an expansion of the B cell compartment, with mature FO B cells accumulating in multiple organs, including the spleen, LNs, bone marrow and peritoneal cavity. Despite this, these animals do not develop lymphoid malignancies, indicating that Bcl-3 over-expression alone is not sufficient to drive lymphomagenesis. Strikingly, MZ B cells are virtually absent from mice expressing transgenic *Bcl3* only in B cells [71,75], providing further evidence that the strength of NF-κB signals controls cell fate decisions in developing B cells in the spleen. Bcl-3^BOE^ mice are also reported to lack MZ B cell precursors and to have fewer B1 B cells in their peritoneal cavity. The increased number of FO B cells in these transgenic mice may be caused by this skewed differentiation, pushing more B cell precursors into the FO B cell pool, but it is also possible that Bcl-3 over-expression alters FO B cell dependence on B cell survival factors, such as BAFF.

Another striking feature of SLOs in *Bcl3*^−/−^ mice is the virtual absence of GCs under steady-state conditions [38,69], and GCs are poorly induced by infection or immunization with experimental model Ags [69,70,76]. As a consequence, these mice have defects in the generation of certain types of “class-switched” Abs that are produced by ASCs generated in GCs. For example, after influenza virus infection, *Bcl3*^−/−^ mice have lower levels of class-switched anti-virus IgG2a Abs, but normal levels of IgM, a type of Ab that is generated independently of GCs and does not require T cell help [69]. Similarly, compared to wild type controls, *Bcl3*^−/−^ mice challenged with heat-killed *Streptococcus* *pneumoniae* have lower serum levels of class-switched Abs specific for the bacteria [76]. Protection from *S* *pneumoniae* infection requires an effective Ab response involving class-switched Abs, and so it is perhaps not surprising that *Bcl3*^−/−^ mice show increased susceptibility to the development of sepsis after infection with live *S* *pneumoniae* [76]. The precise cellular basis for the GC defect in *Bcl3*^−/−^ mice is not clear. However, it is notable that these mice fail to develop dense FDC networks in the follicles after challenge. Instead their FDCs are aberrantly dispersed throughout the white pulp, and their ability to capture Ag is compromised [70]. FDCs are key cells involved in Ag presentation to FO B cells, and they play critical roles in the formation and function of GCs. The FDC phenotype in *Bcl3*^−/−^ mice appears to be a milder form of that seen in *Nfkb2*^−/−^ mice, in which the FDC network is more substantially disrupted: these mice fail to develop any GCs or elicit effective B cell responses [70,77,78]. However, it is not clear if, or how, Bcl-3 influences signalling through the classical or non-canonical NF-κB pathways in FDCs.

The responses of B cells expressing *Bcl3/BCL3* transgenes vary depending on which particular transgenic strain is analysed. When activated by BCR cross-linking *in vitro*, Eµ-*BCL3* splenocytes proliferate more rapidly than wild-type splenocytes. However, when Bcl-3^BOE^ B cells are activated in a similar way they proliferate less well than wild-type B cells, while B cells from another strain with a B cell-specific *Bcl3* transgene behave like wild-type B cells [71]. These differences are difficult to explain, although *BCL3* transgene expression in T cells likely contributes to responses in Eµ-*BCL3* splenocyte cultures: when these cells are removed the enhanced proliferative response to BCR cross-linking is abrogated. Unchallenged Eµ-*BCL3* mice develop increased numbers of GCs, and their serum carries elevated levels of some class-switched Abs (IgG1 and IgA) [74]. However, Bcl-3^BOE^ mice have normal IgG1 levels, a reduced number of GCs, and dampened Ab responses after immunization with T cell-dependent Ags [75]. Again, these differences between strains may be caused by the expression of the *BCL3* transgene by T cells in Eµ-*BCL3* mice. What is notable however about the Bcl-3^BOE^ mice is that despite having increased numbers of FO B cells, these cells appear less able to participate in GC formation. Bcl-3^BOE^ mice also show defective production of IgM and IgG3 in response to immunization with T cell-independent Ag. This is likely due to the paucity of MZ and B1 B cells in these mice, as these B cell subsets are major contributors to T cell-independent Ab responses.

Collectively, these studies demonstrate that endogenous Bcl-3 is critical for the development of B cells and for optimal B cell responses, and that Bcl-3 over-expression can substantially influence B cell behavior. This is presumably because Bcl-3 regulates NF-κB signaling in B cells and/or other cell types critical for B cell function, such as FDCs and T cells. However, the precise molecular mechanisms are not fully understood, and further work is needed to elucidate the details of Bcl-3 function in this key population of adaptive immune cells.

## 7. The Role of Bcl-3 in T Cells

In addition to its role in central T cell tolerance, Bcl-3 has been shown to be involved in the generation of differentiated CD4^+^ T_H_ cells. *In vitro* studies have shown that *Bcl3* deficiency in T cells results in impaired T_H_2 differentiation due to a defect in the expression of GATA-3 [46], the master transcription factor for T_H_2 cell differentiation. Molecular studies have shown that Bcl-3 and p50 are able to bind to κB-like sites within the *gata3* promoter, suggesting Bcl-3 may be able to initiate direct transactivation of *gata3* [46]. These results, together with findings demonstrating a central role for RelB in T_H_1 cell differentiation, have led to the proposition that the balance of Bcl-3 and RelB in the nucleus might, to some extent, determine whether a CD4^+^ T cell differentiates into a T_H_1 or a T_H_2 cell. These studies however, do not exclude other potential mechanistic explanations, such as the recruitment of co-activators to gene promoter regions by Bcl-3, and further investigations are needed to fully clarify Bcl-3’s molecular function in T_H_2 cell differentiation. T_H_2 populations present in *Bcl3*^−/−^ mice also exhibit diminished production of cytokines characteristic for T_H_2 cells, *i.e.*, IL-4, IL-5 and IL-13 [46]. These findings are not surprising considering the reduced expression of GATA-3, as this transcription factor is known to be responsible for the production of these cytokines [79].

Several studies have reported that the *in vitro* differentiation of naïve T cells to T_H_1 [46,80] or T_H_17 cells [80] is not altered by the absence of Bcl-3. Interestingly, however, it has recently been shown that Bcl-3 suppresses the conversion of IFN-γ-producing T_H_1 cells into T_H_17 cells *in vivo* [80]. These experiments were performed using a model of colitis induced by transferring CD4^+^CD25^−^ T cells into *Rag1*^−/−^ mice. Recipients of wild-type T cells succumb to colitis 6–7 weeks after transfer due to the development of IFN-γ^+^ T_H_1 cells, but remarkably, when T cells from *Bcl3*^−/−^ mice were used the recipients remained healthy. When donor T cells were recovered from the spleen, mesenteric LN and colon 7 weeks after transfer, far fewer of the *Bcl3*^−/−^ T cells were IFN-γ^+^ T_H_1 cells, and there was a corresponding increase in less colitogenic IL-17-producing T_H_17 cells. This phenotypic shift was particularly prominent in the mesenteric LN and colon. Conversion to T_H_17 cells was also able to limit the pathogenicity of *Bcl3*^−/−^ T_H_1 cells in experimental autoimmune encephalomyelitis [80], a mouse model of multiple sclerosis. An increase in expression of RORγt, a key transcription factor that controls T_H_17 differentiation [81], and a concomitant decrease in expression of T-bet, the T_H_1 master regulator [82], was found in the IL-17-producing cells, confirming their conversion to the T_H_17 lineage. Enforced over-expression of RORγt is able to make T_H_1 cells develop a T_H_17 cell phenotype [80], and there is evidence that Bcl-3 controls T_H_ cell plasticity by suppressing RORγt production. The *Rorc* gene encodes the RORγt protein, and its promoter contains NF-κB-binding sites that have been shown to interact with the classical pathway subunits c-Rel, p50 and p65 [80,83]. Binding of c-Rel and p65 to these sites induces *Rorc* expression, RORγt production, and T_H_17 differentiation [83]. *Rorc* expression is inhibited in T_H_1 and T_H_17 cells generated from mice expressing a T cell-specific *Bcl3* transgene, and, in the T_H_1 cells, Bcl-3 protein encoded by the transgene is bound to regions of the *Rorc* promoter that contain NF-κB-binding sites [80]. Moreover, *Bcl3* deficiency results in the increased presence of c-Rel on the *Rorc* promoter in T_H_1 cells generated *in vitro*. Thus, in T_H_1 cells, Bcl-3-mediated stabilization of inhibitory p50 homodimers on NF-κB-binding sites in the *Rorc* promoter might be responsible for restricting RORγt production, thereby stabilizing the T_H_1 cell phenotype and restricting conversion to T_H_17 cells.

A role for Bcl-3 in the development of T_FH_ cells has also recently been proposed [84]. In a study by Meguro and colleagues [84], mouse CD4^+^ T cells activated *in vitro* and transduced with *Bcl3*-containing retroviruses were found to express more *Bcl6*, a master regulator of T_FH_ cell differentiation [85], compared to control samples. *Bcl3* retroviruses increased the expression of numerous genes, although other T_FH_ cell markers, such as *Cxcr5*, *Icos* and *Ascl-2,* were not among them. The *Bcl3* retrovirus also enhanced the generation of T_FH_ cells in mice. Conversely, under T_FH_ cell-inducing conditions *in vitro*, reducing the level of endogenous Bcl-3 decreased the number of murine T cells expressing IL-21, a signature cytokine of T_FH_ cells, and suppressed the expression of *BCL6* by human T cells. This study also provides evidence that Bcl-3 can associate with the promoters of the *Bcl6* and *Il21* genes, and, in cell lines, can enhance expression of reporter genes controlled by the *Bcl6* or *Il21* promoters. This suggests that Bcl-3 is directly involved in the transcriptional activation of these T_FH_ genes, although the mechanisms by which it achieves this regulation remain to be defined [84].

In addition to its effect on T_H_ cell differentiation, Bcl-3 has also been reported to act as a survival factor for activated T cells [86,87,88]. These findings are perhaps unsurprising, as it has been known for some time that NF-κB plays a central role in ensuring lymphocyte survival. Ectopic expression of Bcl-3 is able to enhance T cell survival after activation [86,87], and increased cell death is apparent in populations of activated *Bcl3*^−/−^ T cells containing both CD4^+^ and CD8^+^ cells [87]. In this setting, the pro-survival function of Bcl-3 was attributed to its ability to block activation of Bim [87], a pro-apoptotic member of the Bcl-2 protein family, although the molecular mechanisms at work are not clear. CD8^+^ T cells require IL-12 signals at the time of Ag encounter in order to undergo clonal expansion and become fully activated [89]. Bcl-3 expression is up-regulated in CD8^+^ T cells in response to IL-12 stimulation, and *Bcl3*-deficient CD8^+^ T cells are unable to respond to survival signals elicited by IL-12 [90]. It is not known how Bcl-3 controls T cell survival, or how it is induced in activated T cells, but it has been postulated that cytokines produced by DCs activate the transcription factor AP-1, which is known to be involved in the induction of *Bcl3* transcription [91].

Bcl-3 has also been implicated in the control of CD4^+^ T cell anergy during peripheral tolerance. Bcl-3 expression is specifically induced in anergic CD4^+^ T cells and inhibits their production of IL-2 [92]. IL-2 is a potent T cell growth factor that is produced by activated T cells, initiating population expansion and differentiation into effector cells. In this context, Bcl-3 is thought to enhance the binding of p50 homodimers to the IL-2 promoter region, altering the composition of NF-κB dimers in the nuclei of T cells and inhibiting NF-κB-dependent transcription [92]. Bcl-3 has also been shown to inhibit NF-κB-dependent transcription in T cells in response to specific cytokines (IL-4 and IL-9) [55], demonstrating yet another layer of Bcl-3-mediated control of T cell responses. 

Collectively, these studies reveal that Bcl-3 is a context-dependent regulator of T cell responses that can affect the generation of differentiated T_H_ cells, the stability of T_H_ cell phenotypes, the survival of activated CD4^+^ and CD8^+^ T cells, and the reinforcement of T cell anergy. Although much is known about Bcl-3’s involvement in T cell development and function, the molecular mechanisms underpinning these cellular functions are still being revealed, and it will be interesting to see how this area of research develops in the future.

## 8. The Role of Bcl-3 in DC Development and Function

Although not considered adaptive immune cells in their own right, DCs are crucial to the initiation of adaptive immune responses. As mentioned in section 3 above, DCs present Ags to naïve T cells and direct their subsequent differentiation. Bcl-3 is highly expressed in mature DCs [93], and recent work using mice with a DC-specific *Bcl3* deletion (*Bcl3*ΔDC mice) has demonstrated the requirement of Bcl-3 for successful Ag presentation to CD4^+^ and CD8^+^ T cells by DCs, both *in vitro* and *in vivo* [94]. Moreover, *Bcl3* over-expression in DCs enhances T cell priming and proliferation [94]. Bcl-3 contributes to changes in the expression of surface molecules on mature DCs, altering the balance between co-stimulatory and inhibitory factors in favour of activatory signals [94]. However, it is the ability of Bcl-3 to promote DC survival that appears to be critical, allowing optimal DC:T cell interactions to occur, and thereby supporting successful T cell priming. The shortened life-span of activated *Bcl3*^−/−^ DCs may limit the duration of DC:T cell interactions and consequently prevent T cells from receiving the activatory signals required for successful priming. When compared to equivalent cultures of wild type DCs, *Bcl3*-deficient DCs show increased expression of pro-apoptotic genes after stimulation with LPS [94]. This finding is likely to underpin the survival defect of these cells, however, it remains to be determined if Bcl-3 is associated with the promoters of these genes in DCs and, if so, how this controls their activity.

Another recent study has demonstrated that Bcl-3 can also modify cytokine production by activated DCs [95]. Fucose-containing ligands found on extracellular pathogens bind to DC-SIGN, a C-type lectin expressed by DCs. Signalling through DC-SIGN can repress the ability of activated DC to express pro-inflammatory cytokines, including IL-6, IL-12, and IL-23, while enhancing their expression of IL-10 and chemokines capable of attracting T_H_2 cells. During CD4^+^ T cell priming, this shifts differentiation from the T_H_1 to the T_H_2 lineage. Interestingly, the regulation of cytokine/chemokine expression in DCs by DC-SIGN ligands is dependent on Bcl-3-mediated modulation of NF-κB dimer occupancy on cytokine promoters, with DC-SIGN signalling resulting in a shift towards p50 homodimers. This is achieved through the activation of IKK-ε by DC-SIGN, which leads to suppression of CYLD, a deubiquitinase that removes ubiquitin from Bcl-3. As previously mentioned in section 2 above, ubiquitination of Bcl-3 aids its translocation to the nucleus [50]. Consequently, when CYLD is suppressed, ubiquitinated Bcl-3 is able to translocate to the nucleus where it influences which NF-κB dimers are present on cytokine promoters.

Sub-optimal T cell priming results in impaired adaptive immune responses, leaving the host susceptible to infection. Further work by Tassi and colleagues has shown that *Bcl3* expression by DCs is vital for the effective clearance of *Toxoplasma gondii* infection [96]. T cell responses and IFN-γ are critical for protection from this intracellular protozoan, and, unlike their wild-type counterparts, *Bcl3*^−/−^ mice cannot control *T gondii* infection and die within 5 weeks of infection [69]. Remarkably, *Bcl3*ΔDC mice were as susceptible to *T gondii* infection as mice that lacked *Bcl3* in all their cells [96]. Early innate responses to infection were maintained in these animals, but later adaptive responses were compromised: their CD4^+^ and CD8^+^ T cells produced substantially less IFN-γ than wild-type controls at a time during infection when this cytokine is known to be important for protective responses. Although the impact of *Bcl3* loss on DC biology was not specifically explored in this study, defects in DC survival or changes in cytokine production similar to those discussed above could be responsible for the effects observed.

Thus, not only does Bcl-3 control the behaviour of T and B cells during adaptive immune responses, but it also has a key role in ensuring that Ags are presented to naïve T cells, and that T cell-dependent adaptive immune responses are appropriately initiated.

## 9. Bcl-3 and Immunopathology

Since Bcl-3 influences the function of adaptive immune cells, it is perhaps unsurprising that aberrant Bcl-3 expression has been associated with a range of immune dysfunctions. Altered Bcl-3 expression levels have been observed in several autoimmune diseases. A 2010 study identified single-nucleotide polymorphisms in the *BCL3* gene, which predicted reduced Bcl-3 protein expression, as a likely risk factor for Crohn’s disease [97]. A more recent report, however, demonstrates that patients with both Crohn’s disease and ulcerative colitis have elevated expression of *BCL3* compared to healthy individuals [98]. Work using the dextran-sodium sulfate (DSS) mouse model of colitis showed that colitis was associated with a significant increase in *Bcl3* expression, and that *Bcl-3*-deficiency provided partial protection against disease development [98]. *Bcl3*^−/−^ mice demonstrated comparable levels of immune cell infiltration and pro-inflammatory cytokine production compared to wild-type controls, indicating that, in this context, Bcl-3 did not appear to be directly regulating inflammation. Instead, Bcl-3 is thought to play a role in regulating intestinal epithelial cell turnover under inflammatory conditions, with the enhanced epithelial cell proliferation and regeneration present in *Bcl3*^−/−^ mice after disease onset supporting this suggestion [98].

In contrast, *Bcl3* deficiency has been shown to render mice more susceptible to autoimmune diabetes [99]. The absence of *Bcl3* appeared to have no effect on T_H_1 or T_H_2 responses, but it did result in enhanced expression IL-17A, the T_H_17 signature cytokine, indicating that T_H_17 responses were increased [99]. Susceptibility to diabetes is also affected by *c-Rel*-deficiency in mice, which confers resistance to low-dose streptozotocin-induced diabetes [100], indicating that Bcl-3 may be influencing diabetes susceptibility through its role in the classical pathway of NF-κB activation. This work also identified Bcl-3 as a regulator of chemokine gene expression in diabetes, and suggested a possible role for Bcl-3 in T_H_17 cell expansion and/or survival through an IL-23-dependent mechanism. This idea is supported by the work discussed above regarding Bcl-3-mediated stabilization of T_H_1 cell phenotype through the inhibition of RORγt expression [80]. In the absence of Bcl-3, the plasticity of T_H_1 cells is increased, allowing for an enhanced conversion to T_H_17 cells in response to IL-23, and it is possible that such alterations in the properties of diabetogenic T cells aid the development of diabetes.

A 2012 study focusing on the identification of biomarkers predictive for early rheumatoid arthritis (RA) has revealed a correlation between *BCL3* expression by circulating T cells and disease, with *BCL3* identified as one of twelve genes comprising a potential CD4^+^ T cell gene “signature” in RA patients [101]. Similarly, DNA microarray analysis of CD4^+^ T cells from untreated RA patients and healthy controls demonstrated an increased expression of *BCL3* in those with RA [84]. *BCL3* expression decreased after successful treatment with tocilizumab (which blocks the IL-6 receptor), but not after treatment with inhibitors of TNF or CD80/86 [84]. RA patients have elevated levels of circulating IL-6, and this cytokine induces Bcl-3 expression in both mouse and human CD4^+^ T cells [84,102]. It has also been proposed that elevated *BCL3* reflects the increase in circulating T_FH_ cells previously shown to exist in RA patients, which correlates with disease activity [103,104]. Indeed, in RA patients, increased expression of the T_FH_-associated genes *CXCR5*, *ICOS* and *ASCL-2* correlated with elevated levels of *BCL3* expression and higher disease activity [84]. Perhaps, then, IL-6-driven *BCL3* induction enhances the development of T_FH_ in RA.

## 10. Conclusions

Bcl-3 plays important roles in NF-κB regulation, able to either promote or inhibit NF-κB target gene expression depending on the cell type and stimulus received. Expression of Bcl-3 is tightly regulated, and aberrant expression of this IkB molecule results in a range of defects within the immune compartment. Animals with a deficiency in Bcl-3 present with defects in the microarchitecture of their secondary lymphoid organs and disrupted adaptive immune responses, resulting in increased susceptibility to infection. Bcl-3 over-expression detrimentally alters the composition of both the B and T cell compartments, with animals suffering lymphoproliferative disorders. It is clear that the precise contributions of Bcl-3 to adaptive immune cell function are very complex, and despite many important advances the immunological functions of Bcl-3 and still not fully understood. In particular, much more work is needed to define the molecular mechanisms by which Bcl-3 regulates gene expression in immune cells. Importantly, a more in depth understanding of the roles played by Bcl-3 in immune pathologies could, in time, encourage the therapeutic targeting of this important regulator of NF-κB as a way of controlling autoimmune disease and other immunopathologies.

## Abbreviations

The following abbreviations are used in this manuscript:

NF-κB	Nuclear factor κB
BAFF	B-cell activating factor
IκB	inhibitor of κB
Ig	immunoglobulin
TNF	tumor necrosis factor
PTM	post-translational modification
Ags	antigens
TCR	T cell receptor
BCR	B cell receptor
T_regs_	regulatory T cells
FO	follicular
MZ	marginal zone
SLO	secondary lymphoid organ
LN	lymph node
MHC	major histocompatibility complex
DCs	dendritic cells
Ab	antibody
ASCs	antibody-secreting cells
T_H_ cell	helper T cell
T_FH_ cell	follicular T helper cell
GC	germinal centre
mTECs	medullary thymic epithelial cells
SED	sub-epithelial dome
FDCs	follicular dendritic cells
RA	rheumatoid arthritis
IFNγ	interferon-γ
DSS	dextran-sodium sulfate
